# Plutonium isotopes can be used to model soil erosion in Kenya

**DOI:** 10.1007/s10653-024-02084-2

**Published:** 2024-07-29

**Authors:** Sophia Dowell, Olivier Humphrey, Job Isaboke, Thomas Barlow, William Blake, Odipo Osano, Michael Watts

**Affiliations:** 1 Inorganic Geochemistry, Centre for Environmental Geochemistry, British Geological Survey, Nottingham, NG12 5GG UK; 2 School of Geography, Earth and Environmental Sciences, University of Plymouth, Plymouth, Devon PL4 8AA UK; 3 School of Environmental Sciences, University of Eldoret, Eldoret, Kenya

**Keywords:** ^239+240^Pu, Soil erosion, Kenya, MODERN model, Land use change, Tropical soils

## Abstract

Climate change poses an immediate threat to tropical soils with changes in rainfall patterns resulting in accelerated land degradation processes. To ensure the future sustainability of arable land, it is essential to improve our understanding of the factors that influence soil erosion processes. This work aimed to evaluate patterns of soil erosion using the activity of plutonium isotopes (Pu) at sites with different land use and clearance scale in the Winam Gulf catchment of Lake Victoria in Kenya. Erosion rates were modelled at potential erosive sites using the MODERN model to understand small-scale erosion processes and the effect of different management practices. The lowest soil redistribution rates for arable land were 0.10 Mg ha^−1^ yr^−1^ showing overall deposition, resulting from community-led bottom-up mitigation practices. In contrast erosion rates of 8.93 Mg ha^−1^ yr^−1^ were found in areas where steep terraces have been formed. This demonstrates the significance of community-led participation in effectively managing land degradation processes. Another key factor identified in the acceleration of soil erosion rates was the clearance of land with an increased rate of erosion over three years reported (0.45 to 0.82 Mg ha^−1^ yr^−1^) underlining the importance vegetation cover plays in limiting soil erosion processes. This novel application of fallout plutonium as a tracer, highlights its potential to inform the understanding of how soil erosion processes respond to land management, which will better support implementation of effective mitigation strategies.

**Graphical abstract:**

Modelling of soil erosion and deposition patterns using the MODERN model to calculate the depth of soil loss/gain. 
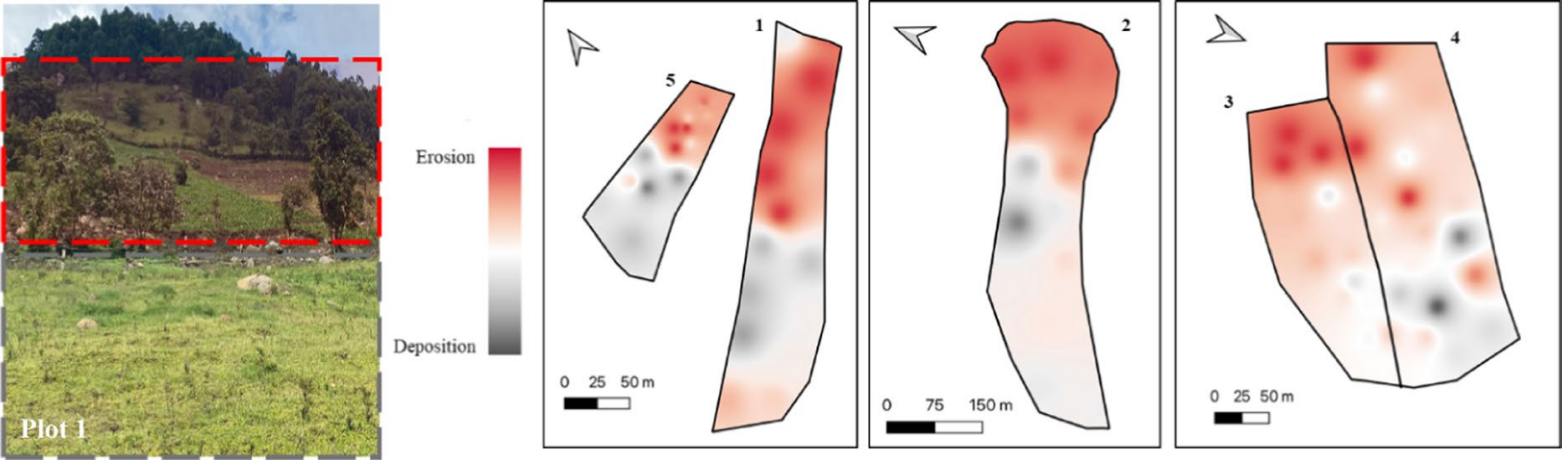

**Supplementary Information:**

The online version contains supplementary material available at 10.1007/s10653-024-02084-2.

## Introduction

Accelerating soil erosion in tropical soils poses an immediate threat to land and water resources in developing nations. With the growing population in tropical Africa and with changes in weather patterns due to global warming, land degradation presents a significant obstacle to the sustainable development of agriculture (Borrelli et al., 2020; Kopittke et al., 2019). Land cover changes and vegetation clearance are primary source of soil erosion, with a growing number of studies recognising soil erosion in tropical soils as a significant environmental concern (Flores et al., 2020; Labrière et al., 2015; Wilken et al., 2021). With the East African Rift valley's steep terrain, high rainfall erosivity, and often insufficient soil cover due to land management and increasingly changes in rainfall patterns, accelerating erosion rates are a major concern (Humphrey et al., 2022; Meshesha et al., 2012; Watene et al., 2021). As a consequence, nutrient deficiency as a result of land degradation in tropical soils poses a substantial hazard, reducing both crop yields and crop protection against disease. This could dramatically increase the risk of global food shortages, negatively impacting both human and animal nutrition (Hickey et al., 2012; Kogo et al., 2021). There has been limited research into the rates of soil erosion in tropical soils and consequently, there is an increasing demand for quantitative data characterising erosion amounts to enhance our understanding of erosion processes so effective mitigation strategies can be implemented. Past studies in Africa have used ^137^Cs (Chappell et al., 1998; Collins et al., 2001; Junge et al., 2010; Pennock, 2000; Quine et al., 1993; Rabesiranana et al., 2016; Ruecker et al., 2008) and ^210^Pb_ex_ (Rabesiranana et al., 2016; Walling et al., 2003), however, these methods have limitations which are notably greater in the southern hemisphere (Dowell et al., 2023a, 2024). This data can also be utilised to validate prediction models, allowing for a clearer understanding of the factors that can influence erosion processes, which can also support the translation of Local Environmental Knowledge (LEK) at the community scale into national policy action (Castro et al., 2020).

Fallout radionuclides (FRNs) such as ^137^Cs and ^239+240^Pu were deposited into the soil as a result of atmospheric nuclear weapons testing in the 1950’s and 60’s, provide an alternative to costly long-term monitoring approaches such as run off plots for soil erosion (Mabit et al., 2014; Parwada et al., 2023; Zapata, 2003). As a result of their capacity to bond strongly to soils, FRN are suited for use as soil erosion tracers and have been successfully utilised across the globe for over 20 years (Alewell et al., 2017; Schimmack et al., 2001, 2002). These techniques work by comparing the inventory (Bq per unit area) of a representative, undisturbed reference site to the inventory at a specific sampling site. By assuming that the loss of radionuclide at the reference site is only due to radioactive decay, soil erosion can be calculated from the loss of radionuclide within the sampling area (Joint FAO/IAEA, 2014). Detailing the subsequent redistribution of the FRN in the soil is a useful method for tracing erosion amounts and patterns within a landscape. Erosion is indicated by a lower FRN inventory than the reference inventory, whereas deposition is indicated by a larger FRN inventory than the reference inventory. In contrast to conventional methods, the FRN method can simultaneously analyse erosion and deposition rates during a single sampling campaign and can be used during a single sampling cycle (Alewell et al., 2017; Meusburger et al., 2018). Analysis of FRNs has proven to be an efficient and time saving way for calculating the redistribution of soil during the past 60 years, with due attention to sources of uncertainty (Parsons & Foster, 2011). These techniques have been utilised to enhance understanding of the resilience and state of agroecosystems worldwide (Alewell et al., 2014; Khodadadi et al., 2021; Lal et al., 2020; Meusburger et al., 2018; Portes et al., 2018; Wilken et al., 2021; Xu et al., 2015).

The environmental cycling behaviour of ^239+240^Pu is comparable to that of ^137^Cs which has been more frequently utilised in the past, and given its advantages over ^137^Cs, this technique has the potential to replace the use of ^137^Cs in tropical soils (Alewell et al., 2017; Xu et al., 2015). Due to the substantially longer half-lives of ^239^Pu and ^240^Pu (24,110 and 6561 years, respectively), about 99% of the original activity is still present in soils, providing a significantly longer future availability than ^137^Cs, which has a half-life of only 30 years (Muller et al., 1978; Schimmack et al., 2002). In addition, recent enhancements to analytical techniques for isotope atom counting, such as ICP-MS/MS, have boosted detection sensitivity (Dowell et al., 2023b; Zhang et al., 2021). Despite having significantly lower environmental activity, six times as many atoms of ^239+240^Pu were distributed from nuclear weapons testing compared to ^137^Cs. Using alpha-particle spectroscopy and accelerator mass spectrometry (AMS), the measurement of plutonium in the environment was limited to a small number of laboratories worldwide (Esaka et al., 2017; Hrnecek et al., 2005; Varga et al., 2007). However, recent advancements in mass spectrometry techniques, such as ICP-MS, have increased the global availability of analysis, hence making the use of Pu isotopes for tracing soil erosion more appealing (Dowell et al., 2023b; Hou et al., 2019; Tiong & Tan, 2019; Xing et al., 2021; Yang et al., 2021).

Radionuclide inventory can be converted into estimated soil redistribution amounts using conversion models. Models which have been frequently used in the past include the Proportional Model (PM), Mass Balance Model (MBM), Profile Distribution Model (PDM) and Diffusion and Migration model (DDM) (Gharbi et al., 2020; He & Walling, 1997; Soto & Navas, 2008; Walling et al., 2011). Although these models are still frequently used the Modelling Deposition and Erosion rates with Radio-Nuclides (MODERN) approach proposed by Arata et al., (2016b) offers several advantages in the modelling of soil erosion by its ability to modify the depth profile of the reference location to simulate natural scenarios such as tillage, erosion, and deposition. This removes many of the assumptions for the reference site, to allow the conversion of isotope inventories into soil redistribution rates, independent of the type of land use (Arata et al., 2016a, 2016b).

Previous studies within tropical Africa, have historically used ^137^Cs and unsupported ^210^Pb to determine soil erosion rates. In Uganda, ^137^Cs was used to measure relatively high soil erosion and deposition rates of -21 to + 25 Mg ha^−1^ yr^−1^ from using a mass balance model (Ruecker et al., 2008). In Zambia, both ^137^Cs and unsupported ^210^Pb determined much lower rates of soil erosion reported between − 5.4 and − 0.3 Mg ha^−1^ yr^−1^, also deploying a mass balance model (Collins et al., 2001; Walling et al., 2003). Furthermore, Wilken et al., (2021) reported the use of the mass balance model incorporating ^239+240^Pu to determine soil erosion in DR Congo, Uganda and Rwanda, with rates between -51.4 and + 20.2 Mg ha^−1^ yr^−1^. In this study, we aimed to determine soil erosion rates at various plots in the Winam Gulf catchment of Lake Victoria in Kenya, each with different land use types and clearance scales with the following objectives: (1); determine the inventory of ^239+240^Pu isotopes at study sites in Western Kenya, (2); relate sample site inventory with reference sites to calculate rates of erosion and deposition using the MODERN model and (3); compare erosion scale according to different land use and management practices.

## Materials and methods

### Study area

**Fig. 1:**
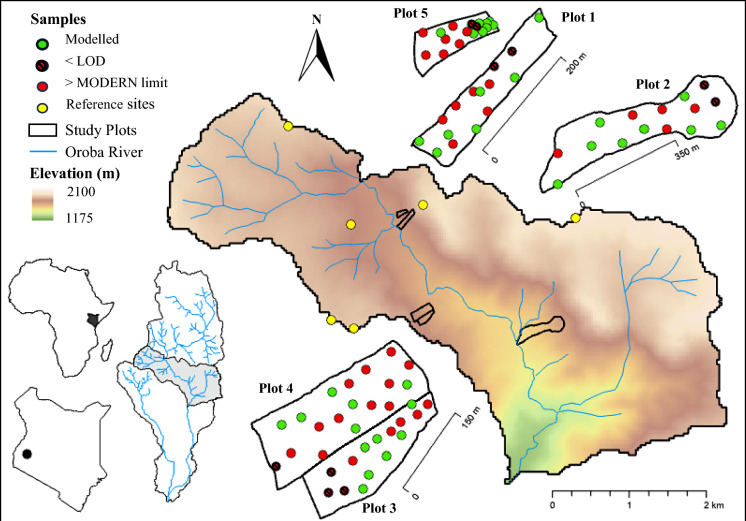
Elevation map of sample location within the upper Oroba river catchment, Nandi County, Kenya. “Not modelled” samples either had undetectable 239 + 240Pu activity concentrations or inventories which exceeded the MODERN confidence limits

This study was carried out in the Oroba valley, located in the escarpment of the rift valley on the border of the Nandi and Kisumu Counties, Kenya (Fig. 1). This valley is characteristic of agricultural soils in the Rift Valley escarpment and provides a representative view of erosion processes in the area. The valley banks onto the Oroba river which eventually drains into the Winam Gulf of Lake Victoria. The soils on the north westerly side of the valley are brown Acrisols which make up plots 1, 3, 4 and 5. The soils within plot 2 are red brown Cambisols. This valley was indicated to be at increased risk of erosion by Humphrey et al., (2022) and it has experienced a range of different management practices alongside continuous land clearance over the past 80 years. For this reason it presented an ideal semi-natural laboratory to evaluate the Pu tracer in a tropical context. Therefore, the valley was selected as an ideal study location to demonstrate the applicability of the plutonium erosion model with MODERN in tropical environments and to predict erosion extent based on different land uses within the Winam Gulf catchment of Lake Victoria.

Many of the originally cleared plots have seen significant erosion events leading to further clearance of the valley to sustain the local people; a perpetual cycle often seen in the tropics. With the valleys steep slopes, increasing rainfall, and changes in land cover, these sites are at high risk of erosion, posing a substantial challenge to the ‘sustainable intensification’ of agriculture in the area (Borrelli et al., 2017, 2020). The reference site data used in this study has been previously published by Dowell et al., 2024. Along the valley, 6 suitable reference sites were selected according to the guidelines set out by Joint FAO/IAEA, (2014). All reference sites were selected according to local knowledge as having been under continuous vegetation cover for the period since deposition of began in the early 1950s and had no visible signs of soil loss or deposition. The applicability of the reference sites for this study were demonstrated in Dowell et al., 2024, where the allowable error was less than 10% and total reference inventory showed good agreement to predicted fallout activity for the area. Samples which had undetectable ^239+240^Pu or had inventories that exceeded the MODERN confidence limits were excluded from the modelling process (Supplementary Table 1).

**Fig. 2:**
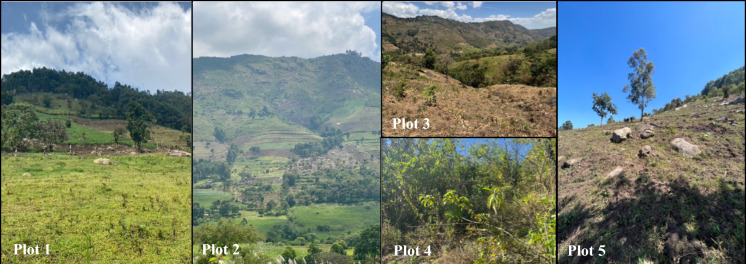
Images detailing the land use, slope and condition of the five study plots

Through evaluating erosion patterns and differences across land management practice within the valley, this cycle of clearance and subsequent degradation of the soils can be further understood and communicated to local stakeholders, to improve our understanding of the effectiveness of mitigation strategies and break the cycle. Within the valley, five study sites were selected according to differing land use, management, and clearance scale (Fig. 2). All 5 plots featured concave slopes which gradually reduced in steepness as you move down the slope.

**Fig. 3:**
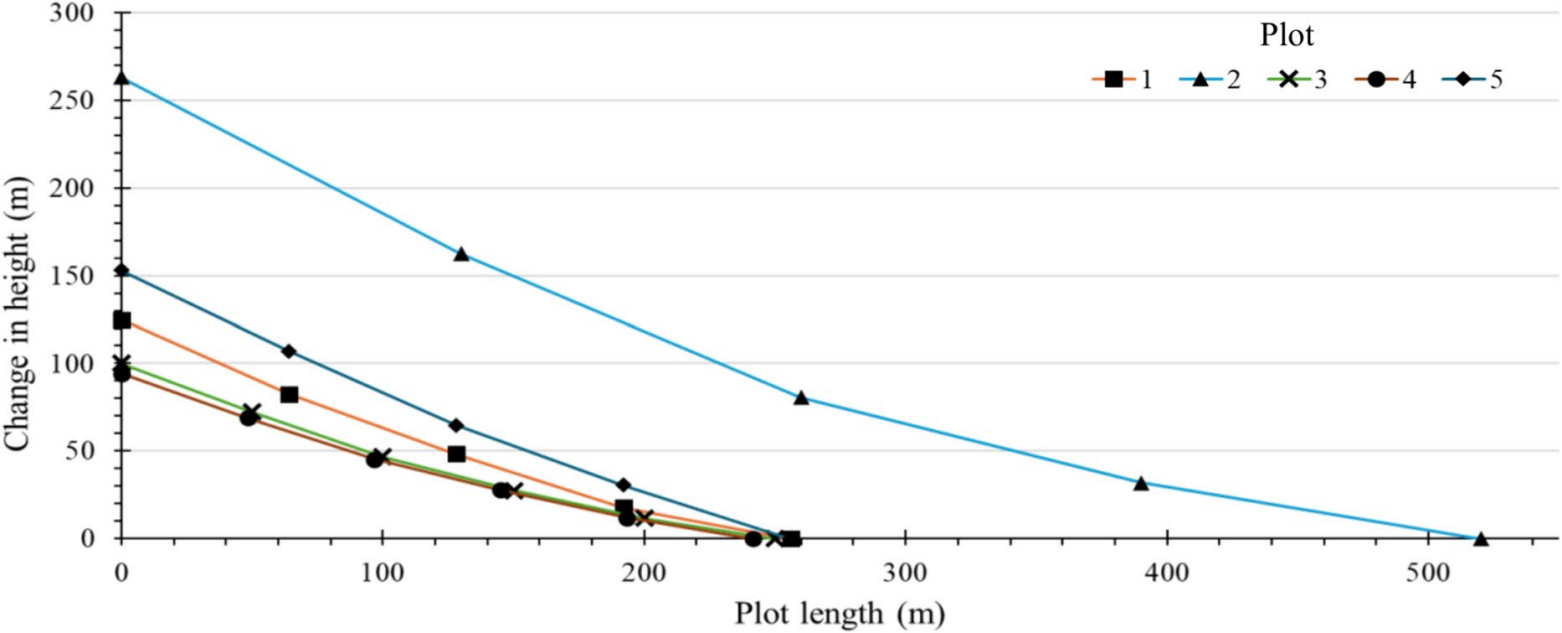
Slope gradients across the 5 study sites

The first four plots were sampled in October 2021 while plot 5 was sampled in January 2023. The first of the four plots was cleared of natural vegetation over 80 years ago. During the past decades farmers have been continually adapting their mitigation strategies based on observational evidence and peer-to-paper Local Environmental Knowledge (LEK) exchange. This includes the use of drainage ditches, cover and companion crops, and crop rotation. The land is hand ploughed using a hoe with a plough depth of approximately 6 cm. The average slope of the plot is 23° with the greatest slopes towards the top of the plot at 33° (Table 1 and Fig. 3). The slope has a flat top with a sheer drop at the edge. The top of plot 1 is an example of a suitable reference location (Dowell et al., 2024).

**Table 1:** Descriptions of study plots selected to demonstrate soil erosion according to differing land use and clearance scale

	Plot 1	Plot 2	Plot 3	Plot 4	Plot 5
Year of first cultivation	1940	1940	2019	N/A	2003
Sampling year	2021	2021	2021	2021	2023
Mitigation practices	Community led adaptation of mitigation based on observational evidence including drainage ditches, cover/companion crops & crop rotation	Terraces formed through the movement of soil into dips with large boulders for the purpose of creating a flat surface to be plough by oxen rather than by hand	Since clearance there has been no mitigation measures implemented	Remains uncleared for the most part	Since clearance the land was not used for cultivation until 2022 and there has been no mitigation measures implemented
Plough technique	Hand ploughed using a hoe	Oxen plough	Hand ploughed using a hoe	None as land remains uncleared	Hand ploughed using a hoe
Sect. "Introduction"					
Slope	33.4°	37.8°	27.2°	27.9°	35.6°
Land use	Grazing	Terrace planting	Maize/Beans crop	Uncleared	Maize/Beans crop
Sect. "Materials and methods"					
Slope	28.1°	32.2°	20.9°	26.2°	33.4°
Land use	Maize/Beans crop	Terrace planting	Maize/Beans crop	Uncleared	Maize/Beans crop
Sect. "Results and discussion"					
Slope	25.6°	20.5°	17.6°	20.2°	28.1°
Land use	Maize/Beans crop	Leafy vegetables	Maize/Beans crop	Grazing	Maize/Beans crop
Sect. "Conclusion"					
Slope	15.5°	13.8°	13.2°	18.1°	25.6°
Land use	Grazing	Grazing	Maize/Beans crop	Uncleared	Maize/Beans crop
Section 5					
Slope	14.0°	9.8°	9.8°	10.6°	15.5°
Land use	Grazing	Sugar cane	Maize/Beans crop	Uncleared	Maize/Beans crop

Plot 2, cleared at the same time as Plot 1, uses terraces designed primarily to allow farmers to plough with oxen. These terraces were created by moving soil into dips in the landscape, which were formed using large boulders. The overall slope of the top half of Plot 2 is very steep, with slopes up to 38°. While terracing typically implies erosion control, in this case, the terraces remained relatively steep, with slopes up to 33°, and were delineated using large rocks. The primary purpose was to create units suitable for ploughing by oxen rather than by hand, as in Plot 1. The site also features rocky outcrops where significant erosion events have occurred in the past. Toward the bottom of the slope, the area is dominated by sugar cane (Saccharum officinarum) with an average slope of 14°.

Plots 3 and 4 are located on the opposite side of the valley and adjoin each other. Plot 3 was cleared to be used for various crops, including maize (*Zea mays*) and beans (*Phaseolus vulgaris* L.) in 2019. Since this clearance there has been no mitigation measures implemented. Plot 3 was re-sampled in January 2023 to assess the temporal changes in soil erosion due to land clearance. On the other hand, plot 4 remains uncleared for the most part and is dominated by native shrubs and grass. The average slope of the two plots is 20°.

Plot 5 had similar topography to plot 1 and is farmed by the same family with the most major difference being the management practices in place. Plot 5 was cleared for cultivation in 2003, however, the land was then not farmed again until 2022 and during this time no land management practices were put in place. The steepest section of the plot was towards the top with slopes up to 36°, whereafter the slope levels off to give slopes similar to plot 1 towards the bottom of 16°.

### Soil sampling design

At each reference site a 30 cm core with a 5 cm diameter was excavated using a bulk density tin by digging a pit and inserting the bulk density tin with a diameter of 5 cm and a height of 3 cm into the pit wall. The reference core comprised of 10 individual stacked samples to determine the ^239+240^Pu depth profile at the reference site where the sampling depth exceeded the depth to which fallout Pu had reached (Dowell et al., 2024). In addition to the reference sites, detailed depth profiles were also collected at an eroded and depositional site within plot one. This depth profile served as an example of Pu migration within the soil profile of sample sites. However, detailed depth profiles were not collected within the other sites. To collect the soils at the sample sites, three cores were collected down to a depth of 60 cm (split into 20 cm increments) using a bucket auger where the depth ensured the entire inventory of Pu had been captured. At each site, the cores were combined and homogenised to create one composite sample representative of each depth increment at that site. The bulk density was determined following sample drying at 30 °C and then the sample was sieved to < 2 mm before reweighing to determine the soil density using the known volume of the bulk density tin. In addition, detailed depth profiles were determined at one erosion site and one deposition site within plot 1 using the same method as for the reference sites collected.

### Analysis of plutonium isotope activity in soil samples

A detailed description of the analytical procedure used for ICP-MS/MS analysis is provided elsewhere (Dowell et al., 2023a). In brief, before analysis, the soils were milled to a fine powder with 50 g of each sample being weighed into a glass beaker and then ashed overnight in a furnace at 550°C. The ashed samples were then placed into a PTFE beaker and spiked with 50 pg of a ^242^Pu spike. To the soil mixture 100 ml of concentrated HNO_3_ was added to leach the Pu isotopes. The soil solution was then heated on a hotplate for 24 h at 70 °C. Subsequently the solution was centrifuged at 3000 rpm for 15 min and the resultant supernatant was then collected and filtered through a 0.45 μm hydrophilic PTFE filter. To the residual soil pellet in the centrifuge tube, 100 ml of water was added and then the pellet was redistributed into the water before being centrifuged again. The additional supernatant was then filtered and added to the sample, adjusting the concentration of HNO_3_ to 8 M. To convert the oxidation state of Pu to IV for separation, 4 g of NaNO_2_ was added to the leachate solution. The Pu isotopes were then separated from the matrix and interfering isotopes using a column containing TEVA resin. The high overall spike recoveries using the method between 81 ± 15% (n = 75), demonstrate high chemical recoveries using the TEVA method. Within each analytical batch two silica sand column blanks (20 g) and CRM sample IAEA 384 (0.5 g) were also prepared to determine detection limits for the method (0.108 ± 0.105 pg kg^− 1 239+240^Pu; n = 32) and assess the accuracy and precision of the separation (114 ± 12 Bq kg^−1^ measured; n = 36 versus certified value of 108 ± 13 Bq kg^− 1^).

The ^239+240^Pu isotope concentrations in the soil were then analysed using ICP-MS/MS with O_2_ gas in the collision-reaction cell to mass shift Pu isotopes away from any ^238^U^1^H^+^ interferences (Agilent 8900, Agilent Technologies, Japan). The sample was introduced using an Agilent IaS micro-autosampler and a Cetac Aridus II desolvating nebuliser (Teledyne CETAC Technologies, Omaha, USA). The instrument was auto-tuned using the Agilent Masshunter software using a 1 μg kg^− 1^ tune solution (SPEX CertiPrep #CL-TUNE-1) for general performance (Dowell et al., 2023a). For samples which had ^239+240^Pu below the detection limit the reported values were calculated according to the limit of detection divided by 2 (0.09 pg Kg^−1^). Concentrations of Pu (pg kg^− 1^) were then converted to mass activity using the specific activity for each Pu isotope.

### Quantitative model for estimating soil *erosion* rates

The mass activities (Bq kg^− 1^) of the FRNs were first converted into areal activities (Bq m^−2^) using the measured mass depth of the < 2 mm soil fraction (kg m^− 2^). Total inventories at each of the sites were then calculated from the sum of total ^239+240^Pu areal activities down the depth profile. Total inventories determined at the sampling sites were then converted into estimated rates of erosion by using the Modelling Deposition and Erosion rates with Radio-Nuclides (MODERN) model (Arata et al., 2016a, 2016b). Soil erosion and deposition derived from the MODERN model are expressed as rates in terms of the thickness of the soil layer impacted by soil redistribution processes. This is achieved by matching the complete inventory of the sample site with the depth profile of the reference site to calculate the depth of soil loss or gain. The point of intersection of the sample on the depth profile of the reference site indicates the model's solution which can then be converted to erosion or deposition. The MODERN model offers advantages over other modelling techniques as it allows for the simulation of different agricultural activities by modelling erosion, and deposition at the reference site, to ensure the reference and sampling sites are comparable to one another (Arata et al., 2016a, 2016b).

In this study, two different adaptations were applied to the reference site depth profile of ^239+240^Pu inventory to account for both erosion and deposition scenarios of the studied sites. For eroded sites (total inventory < reference inventory) 10 additional smoothed layers were added to the reference profile to where an exponential smoothing of FRN inventories is simulated to a new depth of 60 cm. This allows the MODERN model to find a solution when the sample site has a FRN inventory less than the FRN inventory of the last layer of the reference profile (Arata et al., 2016b). The second adaptation simulates an additional six depositional layers added to the top of the depth profile to model deposition at the sites (total inventory > reference inventory). These additional layers were created using of an average of the top two reference site layers, equivalent to the plough depth of 6 cm. Where the inventory of ^239+240^Pu exceeded the modelled reference inventory with additional layers, the sample was not modelled as the total inventory exceeded the MODERN confidence limits (*Supplementary Table 1*). This technique is based on the following assumptions: (1) the plutonium isotopes in the research region originate from global nuclear weapon fallout; (2) the reference site has the same depth distribution of ^239+240^Pu; and (3) deposition at sample sites originates from the plough depth of the reference sites.

The MODERN model provides results in terms of cm of soil losses/gains (*x*’). These results can then be converted to soil erosion/deposition rates as Mg ha^−1^ yr^−1^ (Y) using the following equation:

$$Y = 10 \times \frac{{x}^{\prime}\times xm}{d({t}_{1}- {t}_{0})}$$

where *x*m is the mass depth of the soil at the sample site (kg m^−2^), d is the entire depth increment measured at the sampling site, t_1_ is the year of sampling (yr), and t_0_ (yr) is the year of the main radionuclide fallout; typically, 1963 for ^239+240^Pu (Arata et al., 2016a, 2016b).

## Results and discussion

### Depth distribution of ^239+240^Pu in soils

**Fig. 4:**
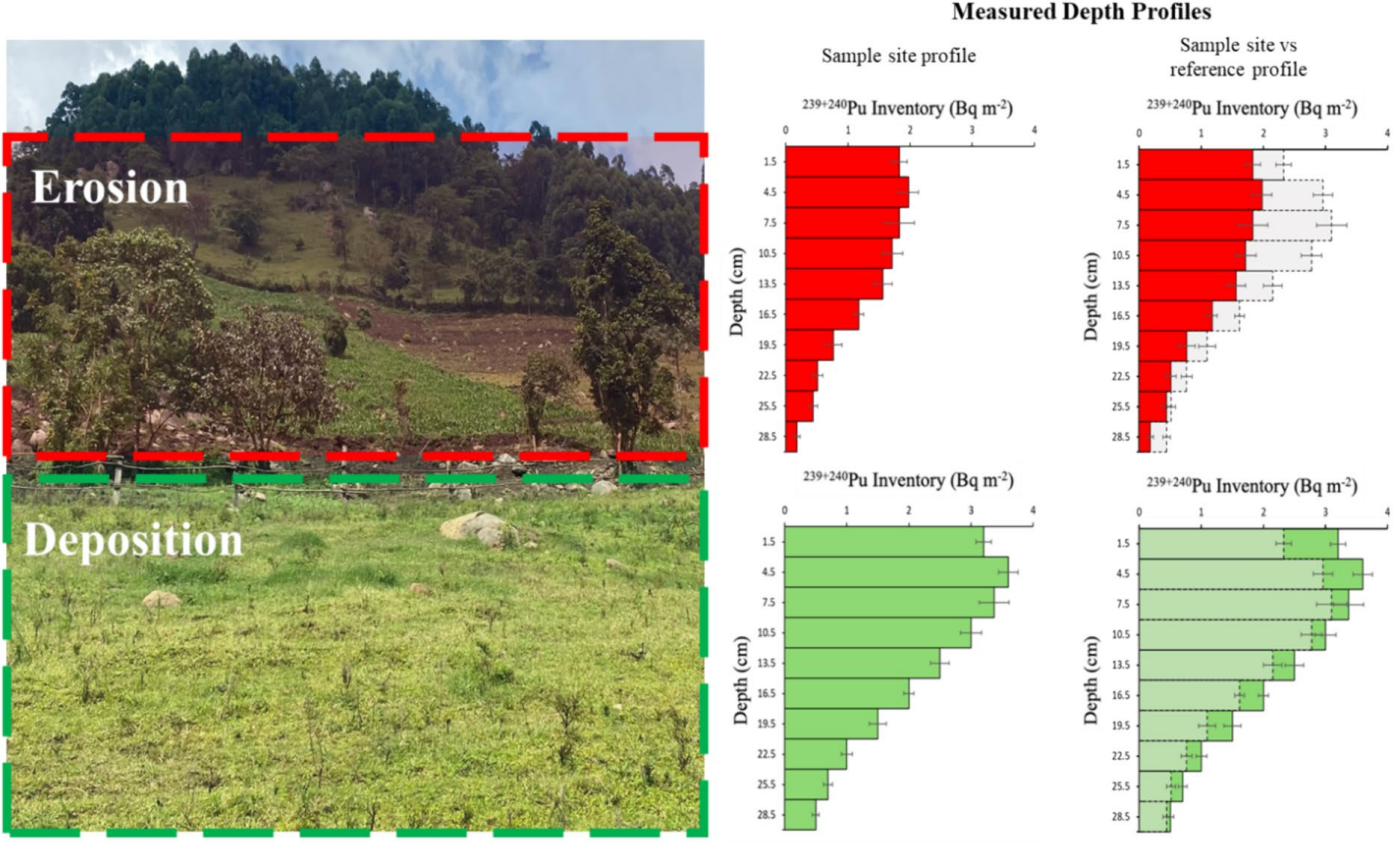
Depth profiles of eroded and deposited soil patterns compared to the depth profile of the reference sites within plot 1

The coefficient of variation and allowable error (as proposed by Sutherland, (1996)), for the ^239+240^Pu reference sites was 11%, and 9%, respectively, meeting the requirement to be used as a suitable reference inventory for the site (Dowell et al., 2024). Figure 4 shows the average depth distribution of ^239+240^Pu at the reference sites as well as an example depth profile of an erosional and depositional site collected within plot 1. This Figure serves as an exemplar depth profile within the study area, however profiles were not taken within the other plots as a detailed depth profile is not required to model soil erosion rates with MODERN (Arata et al., 2016a). The reference sites follow a polynomial function with the maximum areal activities found between a depth of 6–9 cm. Thereafter the areal activities at the reference site decreased exponentially. This has been demonstrated previously as a common pattern of depth distribution, which can be explained by the downward migration of ^239+240^Pu isotopes in the soil subsequently to the main fallout from nuclear tests in the 1950/60 s (Alewell et al., 2014, 2017; Lal et al., 2013; Zhang & Hou, 2019). The ^239+240^Pu areal activities in the reference sites’ surface soils (0–6 cm) of the reference sites range from 3.82 to 5.75 Bq m^−2^ with total inventories ranging from 13.76 to 21.01 Bq m^−2^. The mean inventory of the six reference sites was 18 Bq m^−2^ which is consistent with the fallout estimations by Hardy et al., (1973) and Kelley et al., (1999) for the study region of 19.2 Bq m^−2^, further supporting the validity of the reference site measurements.

Depth profiles at the erosion and deposition sites within plot 1 showed similar patterns with maximum areal activities found between 3 and 6 cm. The top three layers of the depth profile show a similar areal activity relating to the plough depth of 6 cm which has resulted in mixing of the soil layers overtime. Thereafter the depth profiles of ^239+240^Pu at the sampling sites decreased exponentially. The increase in total inventory at the deposition site and the increased areal activity at 30 cm shows the addition of soil layers to the top of the profile. Similarly, this can also be seen for the erosional site where the areal activity at 30 cm suggests that soil layers have been lost towards the top of the profile. The total inventories for the erosion and deposition sites collected within plot 1 were 11.98 Bq m^−2^ and 22.37 Bq m^−2^ respectively. The isotope ratio of ^240^Pu/^239^Pu was also measured alongside the ^239+240^Pu activities for each sample where all sampling sites had a ratio of 0.18 ± 0.02 which agrees with the stratospheric global fallout ratio of 0.18 ± 0.014 (Kelley et al., 1999).

### Inventory and distribution of plutonium in soils

**Fig. 5:**
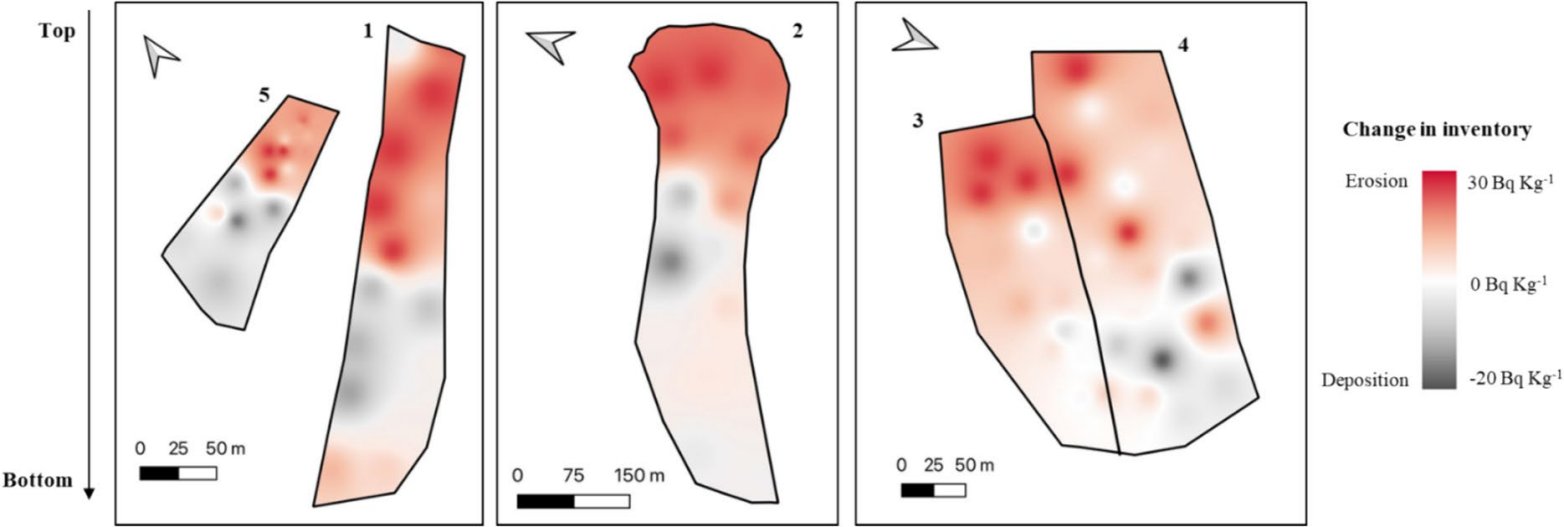
Erosion sensitivity map of study sites within the Oroba Valley, Nandi County, Kenya

**Fig. 6:**
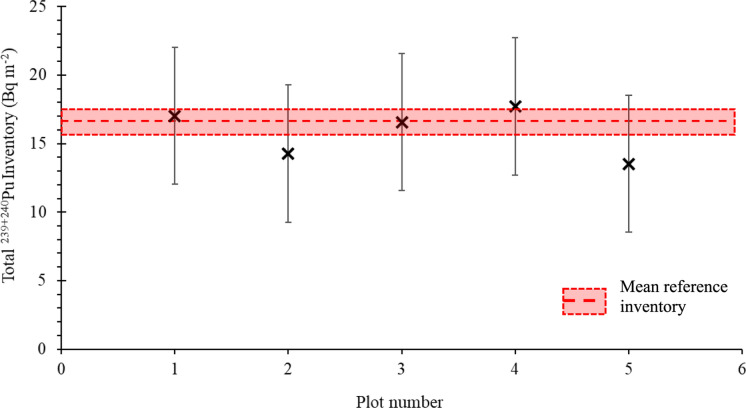
^239+240^Pu mean inventory, where uncertainty is standard deviation, of sample sites compared to the reference site

Within plot 1 the difference between inventory and reference site ranged from -33% to 30% with three sites showing a negative change in inventory indicating erosion with total inventories of 11.98—15.13 Bq m^−2^ and two sites showing a positive change in inventory indicating deposition with total inventories of 21.58–23.49 Bq m^−2^ (Figs. 5 and 6). Plot 2 which was cleared at the same time as plot 1 but with engineering of terraces to create units for ox ploughing saw a much greater range of inventory difference to reference from − 76 to 26%. Within the plot, a total of seven sites showed a negative change in inventory (4.23–17.89 Bq m^−2^) and two showed a positive change (21.28–24.46 Bq m^−2^). Most of the eroded sites (71%) were found within the terraces towards the top of the plot where the steepest topography is found with the depositional sites being found towards the bottom of the plot where sugar cane is grown, which was less steep.

Within plot 5 the difference in inventory ranged from − 63 to 5% with total inventories between 6.67 and 18.82 Bq m^− 2^. The lowest inventories were found towards the top of the plot with a gradual increase towards the bottom of the plot.

Plots 3 and 4, located adjacent to each other, showed similar soil redistribution patterns of soil redistribution but plot 3 had notably greater amounts compared to plot 4. Plot 3 had inventory changes between − 22 and 9% with five sites showing a negative change in inventory (14.01–17.14 Bq m^−2^) and two sites showing a positive change in inventory (18.63–19.55 Bq m^−2^). Plot 4 had a similar range of inventory changes from − 22 to 16% with two sites showing erosion (14.10–16.27), one with a similar inventory as the reference site (17.93 Bq m^−2^) and three sites showing deposition (18.40–20.81 Bq m^−2^). With these two sites being historically very similar, the main variation being that plot 3 was cleared in 2019 while plot 4 remains uncleared, demonstrating the increase in erosion as a result of clearance and land use change.

### Comparison of soil *erosion* rate estimates

The soil redistribution rates, determined using the MODERN model, for each plot are shown in Table 2*.* The highest soil erosion rate was found within the terraced area of plot 2 where estimates of erosion were between 5.44 and 9.41 Mg ha^−1^ yr^−1^. This area had the steepest hill slopes and towards the bottom of plot 2 where slopes were much more moderate redistribution rates were between -5.44 and 8.07 Mg ha^−1^ yr^−1^. In contrast plot 1 which was cleared at the same time showed redistribution rates between -4.41 and 6.52 Mg ha^−1^ yr^−1^. Within plot 5 soil redistribution rates were between -11.20 and 6.46 Mg ha^−1^ yr^−1^.

**Table 2:** Net contribution of soil redistribution rates at sample sites within the Oroba valley, western Kenya (± σ)

	Plot 1	Plot 2	Plot 3	Plot 4	Plot 5
Year of first cultivation	1940	1940	2019	Uncleared	2003
Sampling year	2021	2021	2021	2021	2023
Mitigation practices	Community led	Terraces	None	None	None
Number of sampling sites	7	9	7	6	8
Soil redistribution depth (cm)	0.60 ± 8.30	− 2.89 ± 11.31	− 0.77 ± 3.40	1.26 ± 3.91	− 4.29 ± 7.06
Soil redistribution rates (Mg ha^−1^ yr^−1^)	0.30 ± 4.82	− 1.66 ± 6.50	− 0.45 ± 1.96	0.72 ± 2.25	− 3.58 ± 5.89

Plots 3 and 4 showed varying rates of soil erosion with plot 3 showing a greater overall extent of erosion compared to plot 4. Plot 3 saw redistribution rates between − 2.12 and 2.76 Mg ha^−1^ yr^−1^ while plot 4 had rates between − 2.06 and 4.02 Mg ha^−1^ yr^−1^. This likely a result of deposition occurring within the dense native shrubs within the area due to higher infiltration capacity and reduced overland flow energy. The re-sampling of plot 3 shows the increasing rates of soil erosion as a result of land clearance. Erosion rates had increased up to 4.55 Mg ha^−1^ yr^−1^ in 2023 from 2.12 Mg ha^−1^ yr^−1^ in 2021 in some areas and towards the bottom of the plot deposition had increased to 5.47 Mg ha^−1^ yr^−1^ from 2.76 Mg ha^−1^ yr^−1^. Overall, within the plot the rates of erosion were 0.37 Mg ha^−1^ yr^−1^ higher which is equivalent to an additional half a tonne of soil loss within the area of plot 3 every year.

The rates of erosion determined within the valley are comparable with reported values using ^137^Cs and unsupported ^210^Pb in Zambia, with erosion rates between -5.4 and -0.3 Mg ha^−1^ yr^−1^ (Collins et al., 2001; Walling et al., 2003). The only other study using ^239+240^Pu in tropical African soils found rates found much greater values for both erosion and deposition with rates between -51.4 and 20.2 Mg ha^−1^ yr^−1^.

**Fig. 7:**
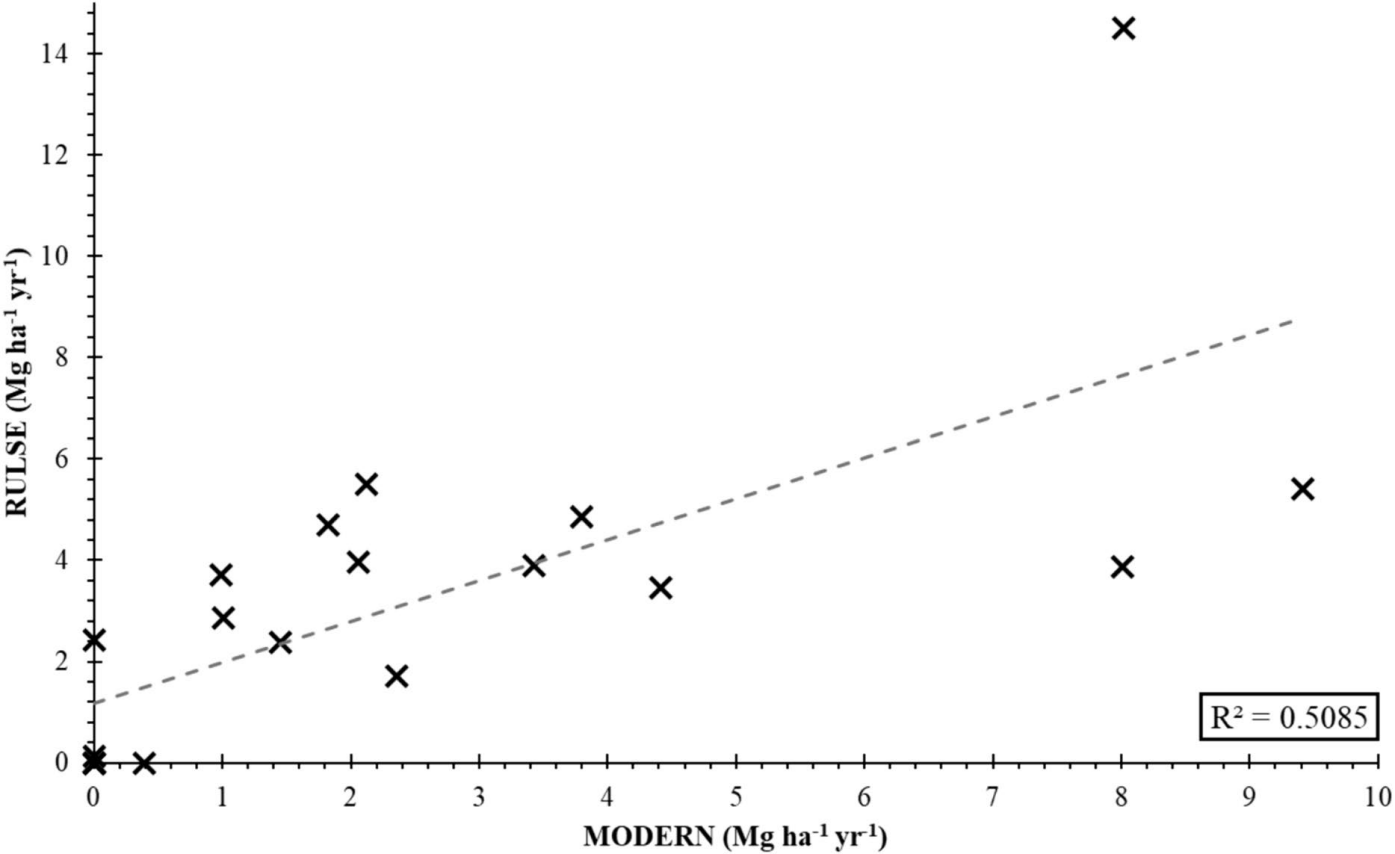
Comparison of predicted soil erosion rates calculated using the RUSLE and MODERN (^239+240^Pu) models within the Oroba valley

Rates of soil erosion within the Winam Gulf have previously been estimated using the RUSLE (Revised Universal Soil Loss Equation) model by Humphrey et al., (2022) with estimated erosion rates between 0 and 14.50 Mg ha^−1^ year^−1^ within the Oroba valley. The overall erosion rates determined using ^239+240^Pu and the MODERN model were 2–3 times lower than the rates calculated using the RUSLE model. However, RUSLE does not take into consideration deposition within the site which skews the results in favour of erosion processes. Furthermore, the RUSLE prediction calculated by Humphrey et al., (2022) did not include the P factor (support practice layer), which could lead to the model overestimating the loss rates as seen. Figure 7 shows the relationship between rates predicted using the RUSLE model and the ^239+240^Pu with MODERN model. Additionally, the RUSLE model demonstrated the potential to be reliable to aid with the selection of appropriate reference sites. Overall, the predicted rates of erosion at the selected reference sites were 0.5 Mg ha^−1^ yr^−1^ with 4 out of the 6 reference sites having a predicted rate of 0 Mg ha^−1^ yr^−1^.

### Land use and management effect on soil *erosion* rates

Due to the relatively small sampling area (40 km^2^) the plots have experienced historically similar rainfall and weather patterns. Across the different plots, the greatest rates of erosion were observed within plot 2 in the areas where the land had been terraced to support ploughing of larger scale land parcels. These terraces are primarily formed by moving large boulders that demark the land parcels but still have slopes up to 33°. Soil movement within these plots may be related to the movement of soil to create the terraces, however, while movement of soil could over time lead to formation of more level terrace surfaces in line with slow forming terrace principles, the net loss of trace inventory from these slopes implies material is still being transferred downslope. This has implications for soil health in the long term possibly resulting from the effects of tillage erosion (Kagabo et al., 2013; Kraemer et al., 2019). This is also demonstrated by the large areas of the land have been denuded and are no longer productive where there is a visible exposure of the bedrock.

In comparison, Plot 1 which was cleared around the same time with similar slopes of 33° saw much lower rates of soil erosion. The main difference between the two plots is that in Plot 1, the farmers have implemented various mitigation strategies. These include using drainage ditches to channel runoff water away from the crop beds and practicing crop rotation combined with mixed pastoral farming, which adds organic matter and enhances soil resilience (Joshi et al., 2021; Kimaro et al., 2012). The farmers are very active in preserving the land which is reflected by the low rates of erosion, despite the severe weather conditions and steep slopes. Additionally, the relatively higher rates of soil erosion seen within plot 5 where no land management practices are in place reiterates the import role soil mitigation practices play in the limitation of land degradation. The effectiveness of community engagement within plot 1 shows the importance it has on implementing successful change. The low overall rates of erosion suggest that the area could be potentially sustainable for agriculture with the correct management of the land.

Plots 3 and 4 show how land clearance affects the acceleration of soil erosion rates within the valley. With plot 3 being cleared in 2019 and plot 4 remaining uncleared the two plots model the change in soil redistribution as a result of erosion. The farmers managing plot 3 are not using any mitigation practices and reported in 2023 that the soil is progressively becoming less productive. This observation is supported by the increased rates of erosion determined in 2023 of 0.82 Mg ha^−1^ yr^−1^, with rates in some areas reaching 5.47 Mg ha^−1^ yr^−1^ (Table 3). Overall, the soil in this plot is more eroded compared to Plot 4, which primarily exhibits deposition processes. This increased erosion is likely due to soil redistribution from runoff originating from both Plot 3 and the cleared land above the uncleared area. This demonstrates the effectiveness of intact vegetation cover in preventing extensive soil redistribution. The data indicates the importance of strengthening the resilience of complex soil erosion challenges to achieve change towards a more sustainable future for tropical soils. Additionally, the differences in overall erosion between plot 1, 2, 3 & 5 (all cleared and used for farming) indicate how even with clearance it is possible to mitigate against soil erosion processes with the correct type of mitigations in place such as drainage ditches and crop rotation. Empirical estimates of soil erosion rates, particularly when comparing different management practices, help assess the risk of land degradation and the sustainability of agriculture.

**Table 3:** Changes in the rate of soil erosion within plot 3 between 2021 and 2023

Sampling year	2021	2023
Soil redistribution depth (cm)	− 0.77 ± 3.40	− 0.99 ± 5.25
Soil redistribution rates (Mg ha^−1^ yr^−1^)	− 0.45 ± 1.96	− 0.82 ± 4.37

## Conclusion

Using variation in ^239+240^Pu activities, this work provides the first insight into soil redistribution patterns in Kenya according to different land management and clearance scale. This work supports previous work modelling rates of soil erosion and provides a clearer image of small-scale erosion processes. The MODERN model returned rates of soil redistribution ranging from -8.93 to 7.14 Mg ha^−1^ yr^−1^ with the greatest rates of redistribution likely related to soil movement as a result of the formation of terracing and steep slopes of up to 38°. The lowest overall rates of soil erosion for arable land resulted from small-scale bottom-up mitigation practices indicating the important role that community led engagement plays in the effective control of land degradation processes. The low net contribution of erosion within the sites suggest that the area could be potentially sustainable for agriculture with effective management of the land. The accelerated erosion rates presented relating to the recent clearance of vegetation for arable farming show the important role that intact vegetation cover plays in ensuring the future resilience of tropical soils. To ensure the future sustainability of arable land and aquaculture downstream, it is necessary to address the complex mix of issues that influence the degradation of tropical soils. This work benefits our understanding of the factors which influence soil erosion and is the first step towards supporting the integration of locally designed solutions into policy.

## Supplementary Information

Below is the link to the electronic supplementary material.

Supplementary file1 (DOCX 238 KB)
