# Supplementary material for: Plutonium isotopes can be used to model soil erosion in Kenya
Source: Environ Geochem Health. 2024 Jul 29;46(9):338. doi: 10.1007/s10653-024-02084-2 (PMC11286636; doi:10.1007/s10653-024-02084-2)
Supplement: Supplementary file 1 — Supplementary file1 (DOCX 238 KB) [file 10653_2024_2084_MOESM1_ESM.docx]

# Plutonium isotopes can be used to model soil erosion in Kenya

Sophia M. Dowell ^a,b^, Olivier S. Humphrey ^a^, Job Isaboke ^c^, Thomas S. Barlow ^a^, William H. Blake ^b^, Odipo Osano ^c^, Michael J. Watts ^a*^

## Supplementary

Supplementary Table 1 – Associated sample data (Sample locations, ^239+240^Pu activity and modelling decision)

| **Sample ID** | **Latitude** | **Longitude** | **Activity**  **(Bq kg^-1^)** | **Inventory (Bq m^-2^)** | **MODERN** |
| --- | --- | --- | --- | --- | --- |
| P1_S1_1 | 0.008706 | 34.988239 | 0.10 | 23.49 | Modelled |
| P1_S2_1 | 0.008209 | 34.987842 | < LOD | - | Not Modelled |
| P1_S2_2 | 0.007996 | 34.987582 | < LOD | - | Not Modelled |
| P1_S2_3 | 0.007829 | 34.987878 | 0.05 | 12.61 | Modelled |
| P1_S3_1 | 0.007727 | 34.987505 | 0.11 | 27.89 | Not Modelled |
| P1_S3_2 | 0.007614 | 34.987274 | 0.13 | 30.36 | Not Modelled |
| P1_S3_3 | 0.007330 | 34.987482 | 0.12 | 29.27 | Not Modelled |
| P1_S3_4 | 0.007610 | 34.987377 | 0.05 | 11.98 | Modelled |
| P1_S4_1 | 0.007408 | 34.987004 | 0.13 | 31.41 | Not Modelled |
| P1_S4_2 | 0.007062 | 34.987313 | 0.09 | 21.58 | Modelled |
| P1_S4_3 | 0.007200 | 34.986821 | 0.14 | 33.71 | Not Modelled |
| P1_S4_4 | 0.006974 | 34.986893 | 0.09 | 22.37 | Modelled |
| P1_S5_1 | 0.006885 | 34.986473 | 0.05 | 12.04 | Modelled |
| P1_S5_2 | 0.006710 | 34.986735 | 0.06 | 15.13 | Modelled |
| P1_S5_3 | 0.006839 | 34.986973 | 0.09 | 20.64 | Not Modelled |
| P2_S1_1 | -0.004453 | 35.005352 | < LOD | - | Not Modelled |
| P2_S1_2 | -0.003961 | 35.005022 | < LOD | - | Not Modelled |
| P2_S1_3 | -0.005112 | 35.005496 | 0.02 | 5.77 | Modelled |
| P2_S2_1 | -0.005222 | 35.004650 | 0.02 | 5.79 | Modelled |
| P2_S2_2 | -0.004629 | 35.004773 | 0.03 | 7.17 | Not Modelled |
| P2_S2_3 | -0.004291 | 35.004478 | 0.02 | 4.23 | Modelled |
| P2_S3_1 | -0.004662 | 35.003826 | 0.12 | 29.73 | Not Modelled |
| P2_S3_2 | -0.005220 | 35.003974 | 0.04 | 9.64 | Not Modelled |
| P2_S3_3 | -0.005204 | 35.003343 | 0.10 | 24.46 | Modelled |
| P2_S4_1 | -0.004805 | 35.003019 | 0.15 | 36.42 | Not Modelled |
| P2_S4_2 | -0.005034 | 35.002102 | 0.09 | 21.28 | Modelled |
| P2_S4_3 | -0.005615 | 35.002737 | 0.07 | 16.57 | Modelled |
| P2_S5_1 | -0.005682 | 35.001862 | 0.07 | 17.89 | Modelled |
| P2_S5_2 | -0.005886 | 35.000923 | 0.10 | 23.98 | Not Modelled |
| P2_S5_3 | -0.006769 | 35.000908 | 0.09 | 22.73 | Modelled |
| P3_S1_1 | -0.004365 | 34.989138 | < LOD | - | Not Modelled |
| P3_S1_2 | -0.004066 | 34.989185 | < LOD | - | Not Modelled |
| P3_S1_3 | -0.004338 | 34.989370 | < LOD | - | Not Modelled |
| P3_S2_1 | -0.004309 | 34.989656 | 0.06 | 15.12 | Modelled |
| P3_S2_2 | -0.003903 | 34.989493 | 0.11 | 26.68 | Not Modelled |
| P3_S2_3 | -0.004110 | 34.989728 | 0.06 | 14.46 | Modelled |
| P3_S3_1 | -0.003707 | 34.989684 | 0.08 | 18.63 | Modelled |
| P3_S3_2 | -0.003834 | 34.990001 | 0.00 | 14.01 | Modelled |
| P3_S3_3 | -0.003580 | 34.989864 | 0.07 | 17.14 | Modelled |
| P3_S4_1 | -0.003476 | 34.990068 | 0.11 | 27.77 | Not Modelled |
| P3_S4_2 | -0.003524 | 34.990219 | 0.08 | 19.55 | Modelled |
| P3_S4_3 | -0.003328 | 34.990205 | 0.10 | 23.60 | Not Modelled |
| P3_S5_1 | -0.003067 | 34.990372 | 0.07 | 17.12 | Modelled |
| **Sample ID** | **Latitude** | **Longitude** | **Activity**  **(Bq kg^-1^)** | **Inventory (Bq m^-2^)** | **MODERN** |
| P3_S5_2 | -0.003216 | 34.990455 | 0.10 | 24.05 | Not Modelled |
| P3_S5_3 | -0.003065 | 34.990599 | 0.09 | 22.95 | Not Modelled |
| P4_S1_1 | -0.003977 | 34.988360 | < LOD | - | Not Modelled |
| P4_S1_2 | -0.003793 | 34.988582 | 0.09 | 21.60 | Not Modelled |
| P4_S1_3 | -0.003376 | 34.988445 | 0.06 | 14.10 | Modelled |
| P4_S2_1 | -0.003815 | 34.989057 | 0.14 | 33.95 | Not Modelled |
| P4_S2_2 | -0.003264 | 34.988734 | 0.08 | 18.77 | Modelled |
| P4_S2_3 | -0.003403 | 34.989000 | 0.10 | 24.40 | Not Modelled |
| P4_S3_1 | -0.003274 | 34.989293 | 0.15 | 36.06 | Not Modelled |
| P4_S3_2 | -0.003427 | 34.989537 | 0.09 | 20.81 | Modelled |
| P4_S3_3 | -0.002884 | 34.989194 | 0.07 | 17.93 | Modelled |
| P4_S4_1 | -0.002770 | 34.989448 | 0.17 | 40.77 | Not Modelled |
| P4_S4_2 | -0.003070 | 34.989494 | 0.07 | 16.27 | Modelled |
| P4_S4_3 | -0.003078 | 34.989774 | 0.09 | 21.43 | Not Modelled |
| P4_S5_1 | -0.003090 | 34.990042 | 0.13 | 31.57 | Not Modelled |
| P4_S5_2 | -0.002766 | 34.990035 | 0.19 | 45.22 | Not Modelled |
| P4_S5_3 | -0.002550 | 34.989694 | 0.03 | 8.37 | Not Modelled |
| P4_S6_1 | -0.002287 | 34.990062 | 0.12 | 28.97 | Not Modelled |
| P4_S6_2 | -0.002488 | 34.990334 | 0.11 | 27.44 | Not Modelled |
| P4_S6_3 | -0.002778 | 34.990297 | 0.08 | 18.40 | Modelled |
| P5_S1_1 | 0.008652 | 34.987511 | 0.03 | 6.67 | Modelled |
| P5_S1_2 | 0.008605 | 34.987566 | 0.05 | 11.57 | Modelled |
| P5_S1_3 | 0.008632 | 34.987468 | 0.05 | 12.02 | Modelled |
| P5_S2_1 | 0.008562 | 34.987472 | 0.06 | 13.55 | Modelled |
| P5_S2_2 | 0.008496 | 34.987412 | 0.04 | 10.81 | Modelled |
| P5_S2_3 | 0.008621 | 34.987356 | 0.07 | 16.80 | Modelled |
| P5_S3_1 | 0.008620 | 34.987242 | < LOD | - | Not Modelled |
| P5_S3_2 | 0.008574 | 34.987309 | < LOD | - | Not Modelled |
| P5_S3_3 | 0.008470 | 34.987271 | 0.08 | 18.82 | Modelled |
| P5_S4_1 | 0.008505 | 34.987170 | < LOD | - | Not Modelled |
| P5_S4_2 | 0.008327 | 34.987069 | 0.16 | 38.45 | Not Modelled |
| P5_S4_3 | 0.008580 | 34.986969 | 0.15 | 35.30 | Not Modelled |
| P5_S5_1 | 0.008394 | 34.986854 | 0.17 | 41.79 | Not Modelled |
| P5_S5_2 | 0.008165 | 34.986830 | 0.11 | 26.43 | Not Modelled |
| P5_S5_3 | 0.008492 | 34.986787 | 0.07 | 18.01 | Modelled |
| P5_S6_2 | 0.008489 | 34.986519 | 0.12 | 29.02 | Not Modelled |
| P5_S6_3 | 0.008160 | 34.986563 | 0.14 | 33.66 | Not Modelled |
